# Expired Patents

**Published:** 1907-04

**Authors:** 


EXPIRED PATENTS.

February 25, 1907.

421952. Combined dental separator and matrix. W. H. Marshall, Oxford, Miss.
422165. Dental plate. J. J. Stedman, La Porte, Ind.
422350. Artifical Tooth. O. Lund, Philadelphia, Pa.

March 11, 1907.

423205. Automatic dental plugger. H. C. Ballard, Milwaukee, Wis.
423239. Artifical tooth crown and method of mounting the same. W. H. Gates, Philadelphia, Pa.
423344. Dental drill. A. Beiter, Utica, N. Y.

March 18, 1907.

423467. Artifical tooth crown. E. P. Brown, Flushing, N. Y.
423617. Electrical Device for use in dental operations. C. W. Manker, Nebraska City, Nebr.
423616. Galvanic apparatus for dental surgery. C. W. Manker, Nebraska City, Nebr.

The above lists of patents are furnished by Davis & Davis, Solicitors of American and Foreign Patents, Washington, D. C., and St. Paul Building, New York City.

